# Marburg Virus Disease: Pathophysiology, Diagnostic Challenges, and Global Health Preparedness Strategies

**DOI:** 10.5334/aogh.4671

**Published:** 2025-06-02

**Authors:** Delfin Lovelina Francis, Saravanan Sampoornam Pape Reddy, Anitha Logaranjani, Subramnayam S Sai Karthikeyan, Manish Rathi

**Affiliations:** 1 Saveetha Dental College and Hospital, SIMATS, Saveetha University, Chennai, India; 2 Department of Periodontology, Army Dental Centre (Research & Referral), New Delhi, India; 3 Meenakshi Ammal Dental College and Hospital, Meenakshi Academy of Higher Education & Research (deemed‑to‑be‑university), Chennai, Tamil Nadu, India; 4 Assistant Professor, GITAM Dental College and Hospital, Vishakapatnam, Andhra Pradesh, India; 5 Assistant Professor, Department of Periodontology, Army Dental Centre (Research & Referral), New Delhi, India

**Keywords:** Marburg virus disease, epidemiology, global health

## Abstract

*Background:* Marburg virus disease (MVD) is a highly virulent viral hemorrhagic fever with reported case fatality rates of up to 90%. It is part of the same family as the Ebola virus (*Filoviridae*). MVD, originally identified in 1967 in the context of outbreaks associated with African green monkeys, has been reported sporadically in Africa. Recent outbreaks, including those in Equatorial Guinea and Rwanda, underscore the need for robust preparedness systems and global response.

*Objectives:* This narrative review focuses on the pathogenesis, clinical manifestations, diagnostic challenges and treatment strategies regarding MVD. It also stresses the need for better surveillance, diagnostic capabilities and vaccines to help prepare for future outbreaks.

*Methods:* A comprehensive review of clinical data, epidemiological trends, and diagnostic developments was performed by searching relevant literature in *PubMed*, *Medline* and *Scopus*. The relevant data were extracted from studies on MVD and presented as a narrative review.

*Findings:* MVD primarily affects immune and endothelial cells, resulting in a consequent cytokine storm, coagulopathy, and multi‑organ failure. Early symptoms such as fever, headache and myalgia are nonspecific and can delay diagnosis, as they mimic other infections. Monoclonal antibodies and newer antiviral agents are presently being evaluated for the management of MVD.

*Conclusions:* MVD leads to significant morbidity and mortality, and the high fatality rate, along with the absence of targeted therapies, represents a serious global health threat. Collectively, the establishment of infrastructure for diagnostics, global collaboration, and advanced vaccine development will help bolster the response to MVD outbreaks and thus shorten periods of spiking mortality.

## Introduction

The recent outbreaks of Marburg virus disease (MVD) in Africa, particularly in Equatorial Guinea, underscore a significant global health issue. MVD is classified as one of the most lethal and infectious diseases, with a catastrophic fatality rate of 90%. The Marburg virus outbreaks have revealed substantial weaknesses in health systems and emphasize the pressing necessity for comprehensive worldwide preparedness strategies, akin to those established for Ebola virus [1]. Recent outbreaks have accentuated the need for immediate international support in response operations, as well as the vulnerability of the health systems of certain African nations. The disease was initially recognized in 1967 during epidemics in Belgrade, Serbia, and Germany (Marburg and Frankfurt), and was associated with laboratory research involving *Cercopithecus aethiops* (African green monkeys) from Uganda [2]. Subsequent epidemics demonstrated that the virus may spread zoonotically through contact with wildlife, particularly fruit bats, despite its early association with laboratory conditions. Thereafter, epidemics, as well as isolated events, have been documented in countries such as Equatorial Guinea, Ghana, Kenya, the Democratic Republic of Congo, Guinea, South Africa (in a traveler from Zimbabwe), Uganda and Tanzania [3]. Two isolated instances were recorded involving visitors who traveled to Uganda, where they encountered *Rousettus aegyptiacus* bats in 2008 [4]. The primary cause of new infections remains zoonotic spillover events, and controlling future outbreaks necessitates a thorough understanding of wildlife–human interactions.

The insights gained from the COVID‑19 pandemic underscore the vital significance of early detection, swift responses, and continuous investment in public health infrastructure [5]. The Marburg virus, categorized within the Marburg virus genus, *Filoviridae* family, and order *Mononegavirales*, comprises only a single species named *Marburg marburgvirus* (often referred to as “Marburg virus”) [6]. Phylogenetic analysis of genomic sequences from multiple outbreaks identifies five distinct lineages of the virus, leading to the recognition of two different variants: Marburg virus (MARV) and Ravn virus (RAVV). The human transmission of MARV resembles that of Ebola viruses, including Sudan, Bundibugyo, and Ebola viruses (EBOV). Researchers are particularly interested in the viral structure and replication mechanisms of MARV and RAVV because they share several molecular properties with other filoviruses and may inspire future therapeutic approaches [7].

**Figure 1:**
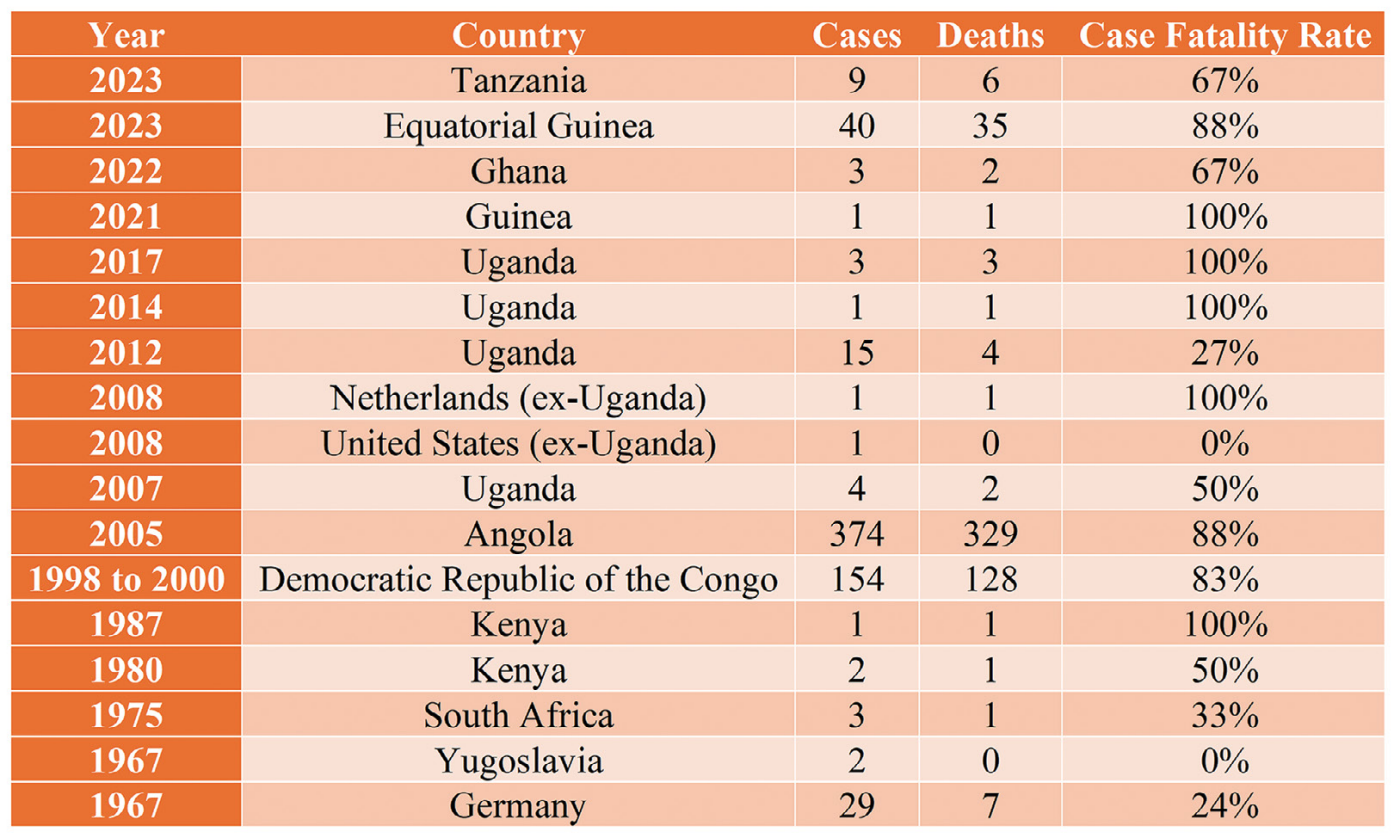
Case fatality rate of Marburg virus.

Identifying the natural reservoirs of MARV has proven challenging due to its intermittent occurrence. However, research has established *Rousettus aegyptiacus* bats as the primary natural reservoir, with evidence suggesting that other bats, such as *Hipposideros caffer*, may also serve as sources of infection [8]. The World Health Organization (WHO) has declared that the Marburg virus has resulted in multiple outbreaks with varying case fatality rates [9]. In 2023, Tanzania reported 9 cases with 6 deaths (67% fatality rate), while Equatorial Guinea recorded 40 cases and 35 deaths (88% fatality rate). In 2022, Ghana experienced three cases with two deaths (67% fatality rate), and Guinea reported a single case with a 100% fatality rate in 2021 [10]. Uganda has faced multiple outbreaks, including 3 cases in 2017, 1 case each in 2014 and 2012, and 15 cases in 2010, with fatality rates ranging from 27% to 100% [11]. Further cases have been reported in the United States and the Netherlands associated with Uganda, alongside substantial outbreaks in Angola (374 cases and 329 fatalities, 88% mortality rate) and Kenya [12]. Early outbreaks in South Africa (1975), Yugoslavia (1967), and Germany (1967) reported lower fatality rates, ranging from 0% to 33% [13]. These data underscore the sporadic nature of Marburg virus outbreaks, their generally high case fatality rates, and the critical need for continued vigilance and preparedness to address this threat (Figure 1).

## Pathophysiology

The pathogenesis of the Marburg virus involves a complex interrelationship between the virus and host immune mechanisms, resulting in severe illness and, frequently, mortality. The virus infiltrates the body via mucosal surfaces or compromised skin following direct exposure to infected bodily fluids, including blood, vomit, or feces. Upon penetration, the Marburg virus predominantly infects immune cells, such as macrophages, monocytes and dendritic cells, in addition to endothelial cells that constitute the lining of blood vessels. The virus utilizes its glycoprotein to bind to cell surface receptors, specifically the DC‑SIGN receptor, on dendritic cells, thereby facilitating entry into the cell. Once inside, the Marburg virus begins to replicate within the host cells. The viral replication cycle disrupts normal cellular functions and causes the host cell to release large amounts of viral particles, leading to the systemic spread of the virus. This widespread replication results in the destruction of infected cells and the release of pro‑inflammatory cytokines, which contribute to a hyperinflammatory response, often referred to as a “cytokine storm”. This inflammatory response can lead to tissue damage, particularly in the liver, spleen, and lymphoid tissues, and contributes to the hemorrhagic manifestations of the disease, including bleeding from various parts of the body [14].

A key feature of the pathogenesis of the Marburg virus is its ability to disrupt the coagulation cascade. The virus damages endothelial cells, increasing blood vessel permeability, fluid leakage, and microvascular clotting, which, in turn, results in disseminated intravascular coagulation (DIC). DIC leads to the consumption of clotting factors, causing excessive bleeding in various organs and tissues. This hemorrhagic tendency is one of the hallmark signs of severe Marburg virus infection, and it can lead to a rapid decline in the patient’s condition [15].

**Figure 2:**
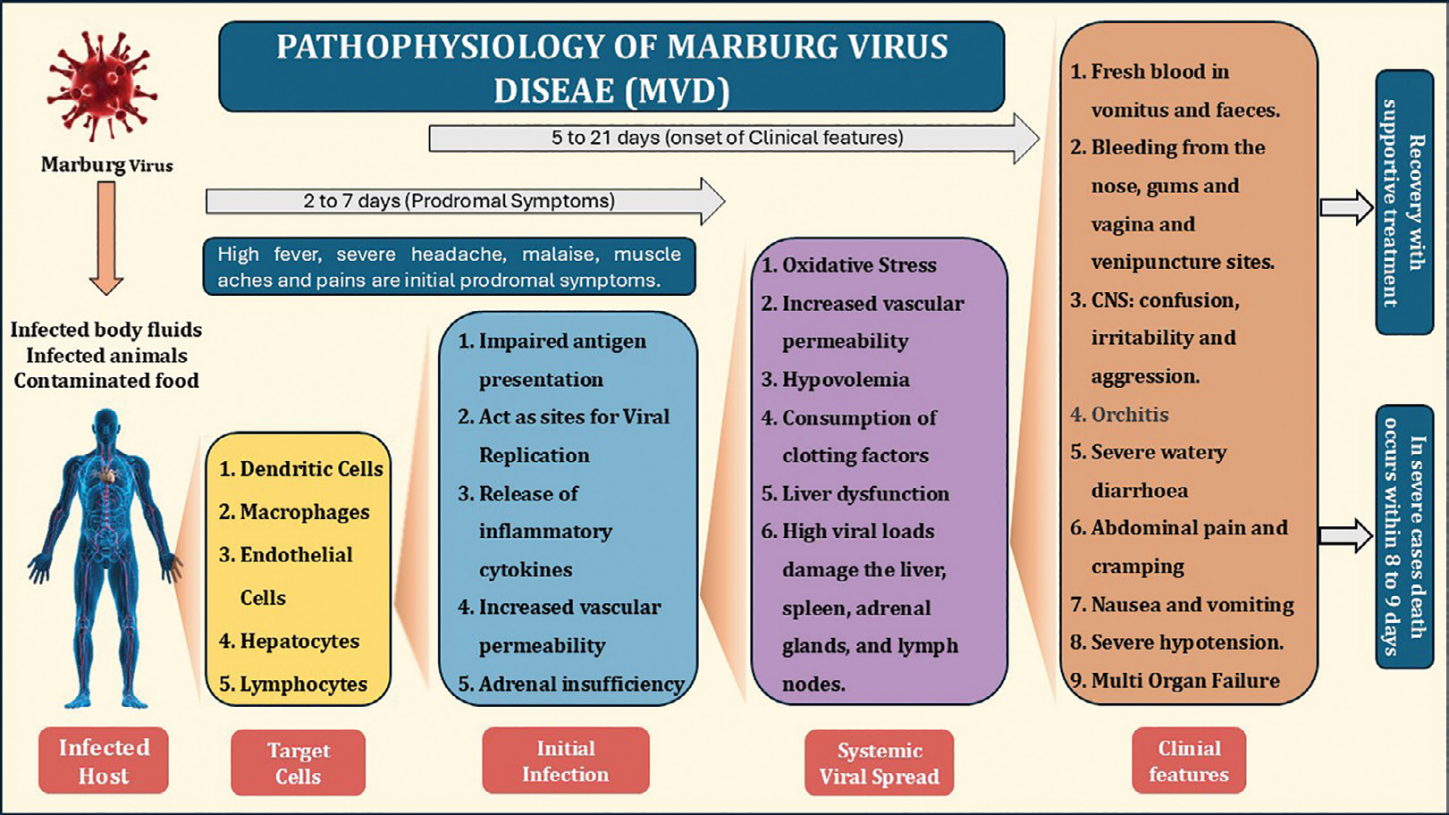
Etiopathogenesis of Marburg virus.

In addition to its effects on the immune and coagulation systems, the Marburg virus exerts a profound impact on the cardiovascular system. Infection results in widespread vascular leakage, leading to decreased blood volume and hypovolemic shock. The subsequent decline in blood pressure and perfusion to vital organs further exacerbates tissue damage. Multi‑organ failure, including liver, kidney, and heart failure, is common in fatal cases and primarily results from viral‑induced damage and the body’s inability to cope with the overwhelming inflammatory response. The immune response to this virus also contributes to disease severity. It suppresses the host’s immune defense mechanisms by inhibiting interferon production, which are key proteins that help control viral infections (Figure 2). This suppression hinders the host’s capacity to initiate a robust immune response, permitting the virus to multiply uncontested and resulting in extended and severe disease [16].

## Diagnosis

Outbreaks frequently strain health systems, delay diagnoses, and limit containment efforts. The delayed identification of Marburg cases during the 2023 Equatorial Guinea outbreak, attributable to inadequate testing facilities, has heightened the potential for regional transmission. Marburg virus disease (MVD) is a critical type of hemorrhagic fever induced by a virus belonging to the *Filoviridae* family and is also associated with the Ebola virus.

The diagnosis of MVD entails a synthesis of clinical evaluation, laboratory analysis, and the elimination of other conditions presenting analogous symptoms. The initial manifestations include chills, fever, headache, malaise, myalgia, and emesis. As the infection advances, patients may develop hemorrhagic manifestations, including gingival bleeding, epistaxis, hematochezia, and ecchymosis. Severe cases may culminate in multi‑organ failure and shock, frequently leading to mortality. Laboratory diagnosis is largely performed through techniques such as polymerase chain reaction (PCR), which identifies viral ribonucleic acid (RNA) in blood specimens, and the detection of the antigen via enzyme‑linked immunosorbent assays (ELISA). Serological assays can also identify antibodies (IgM and IgG) that are specific to the virus. Virus isolation in specialized laboratories can also verify the presence of the Marburg virus.

## Treatment Protocol

MVD was treated with supportive care, as there was no specific antiviral medication available initially. Currently, it is managed with Remdesivir and the glycoprotein‑targeted monoclonal antibody MBP091, which was utilized during the 2024 outbreak of the Marburg virus in Rwanda [17]. The chances of survival for an infected individual dramatically increase with the supportive care provided. Hydration is a critical component of treatment, as symptoms such as fever, diarrhea, and vomiting frequently cause severe dehydration. Rehydration, whether administered orally or intravenously, is vital for maintaining fluid balance. Additionally, maintaining electrolyte balance is essential, as the Marburg virus can disrupt normal physiological functions, leading to imbalances that impair organ function. Oxygen therapy may be required for patients experiencing respiratory distress or hypoxia to ensure adequate oxygen levels in the body.

The management of hemorrhagic symptoms and blood clotting disorders represents a critical component of supportive care. Disseminated intravascular coagulation (DIC), a common consequence of Marburg virus infection, results in abnormal bleeding and clotting. Blood transfusions may be necessary to replenish lost blood and elevate low platelet counts, thereby aiding in the control of bleeding. Coagulation abnormalities must also be meticulously managed to prevent excessive bleeding and ensure patient survival [18].

Although no established antiviral therapy exists, several experimental approaches have been investigated. One promising strategy involves the use of monoclonal antibodies, such as mAb114, which are designed to specifically target and neutralize the Marburg virus [19]. These antibodies can either prevent the virus from infecting host cells or neutralize it once inside the body [20]. Favipiravir, another investigational antiviral, has demonstrated potential efficacy in treating Marburg virus, although its success in clinical application remains under investigation [21]. Ribavirin, an antiviral drug employed for other infections, has exhibited limited efficacy against the Marburg virus in studies [22].

## Plan of Action

The Egyptian fruit bat (*Rousettus aegyptiacus*), recognized as the primary reservoir of the Marburg virus, presents significant challenges for control efforts. Interactions between humans and bats, frequently driven by food scarcity and hunting practices, pose ongoing threats to public health. This situation necessitates community‑oriented initiatives aimed at mitigating human–wildlife interactions and enhancing public health education. Moreover, the absence of an approved vaccine or antiviral medications underscores the critical need for supportive care. Several investigational Marburg vaccines have already been tested in various animal models and are currently in the preliminary stages of clinical trials. For example, vaccines such as the VSV‑EBOV Ebola vaccine, which utilizes a similar viral vector, may provide a framework for the development of Marburg virus vaccines in the future [23]. The WHO and other global health agencies must prioritize funding and equitable distribution strategies for these vaccines once they receive approval.

To prevent future outbreaks, global health stakeholders must strengthen regional diagnostic capacities, enhance surveillance systems, and foster transboundary collaborations. Governments must also invest in health workforce training to improve outbreak detection and responses. Community engagement is a critical component for ensuring that preventive measures align with cultural practices and gain local support. Robust funding mechanisms, such as those initiated during the COVID‑19 pandemic, should be institutionalized for all emerging infectious diseases, including MVD. The world cannot afford to adopt a reactive approach to public health crises, and proactive measures are the cornerstone of sustainable health security. The resurgence of MVD reminds us that infectious diseases are a global threat. Bridging gaps in preparedness, particularly in vulnerable regions, is essential for reducing the human toll of outbreaks and protecting public health on a global scale.
